# HEBE, a novel positive regulator of senescence in *Solanum lycopersicum*

**DOI:** 10.1038/s41598-020-67937-z

**Published:** 2020-07-03

**Authors:** Sara Forlani, Carolina Cozzi, Stefano Rosa, Luca Tadini, Simona Masiero, Chiara Mizzotti

**Affiliations:** Department of Biosciences, Università degli Studi di Milano, Via Celoria 26, 20133 Milan, Italy

**Keywords:** Photosynthesis, Molecular engineering in plants, Plant cell death, Plant molecular biology, Plant physiology

## Abstract

Leaf senescence and plant aging are traits of great interest for breeders. Senescing cells undergo important physiological and biochemical changes, while cellular structures such as chloroplasts are degraded with dramatic metabolic consequences for the whole plant. The possibility of prolonging the photosynthetic ability of leaves could positively impact the plant’s life span with benefits for biomass production and metabolite accumulation; plants with these characteristics display a stay-green phenotype. A group of plant transcription factors known as NAC play a pivotal role in controlling senescence: here we describe the involvement of the tomato NAC transcription factor *Solyc12g036480*, which transcript is present in leaves and floral buds. Since its silencing delays leaf senescence and prevents plants from ageing, we renamed *Solyc12g0364 HḖBĒ,* for the Greek goddess of youth. In this manuscript we describe how *HEB* downregulation negatively affects the progression of senescence, resulting in changes in transcription of senescence-promoting genes, as well as the activity of enzymes involved in chlorophyll degradation, thereby explaining the stay-green phenotype.

## Introduction

Senescence is crucial for plant fitness^1^ and it is a trait of great interest for breeders, since premature senescence can affect crop yield, post-harvest storage and quality^2^. Plant aging can be induced either by endogenous signals or by environmental stresses triggering controlled disassembly and disintegration at cellular and tissue levels, which ultimately affects the whole organism^3^. Organ senescence can be achieved through the activity of relevant cell structures and metabolic processes, such as organelle dismantling and chlorophyll breakdown, a phenomenon which causes the macroscopic leaf color changes observed in green plants. Moreover, previously accumulated macromolecules (i.e. proteins, lipids, nucleic acids and pigments) are also degraded and their products are relocated into sink tissues or organs^4–6^. In annual plants, nutrients are transferred to fruits or seeds, in perennial ones to stems and roots ^7^.

Organ senescence is also accomplished by cell wall modifications, phytohormone fluctuations, dismantling of macromolecules and activation of specific genes; these processes occur during leaf and petal senescence as well as during fruit ripening. The global regulation of these developmental programs involves several players, such as transcription factors, sugars, polyamines and hormones (for reviews see Wojciechowska et al.^8^ and Forlani et al.^9^). The genetic program behind senescence is highly complex and regulated at transcriptional, post-transcriptional, translational and post-translational levels^3^. Several studies have demonstrated the relevance of epigenetic mechanisms in the control of leaf senescence and fruit ripening^10–12^.

NAC transcription factors (NAM No Apical Meristem, ATAF1/2 and CUC Cup-Shaped Cotyledon) play a pivotal role in leaf senescence. This family is one of the largest plant-TFs families and comprises 101 members in *Solanum lycopersicum*, 138 in *Arabidopsis thaliana*, 158 in *Oryza sativa* ssp. *indica*, and more than 400 in *Brassica napus* (The PlantTFDB, https://planttfdb.cbi.pku.edu.cn/). NAC proteins are activators and/or repressors of gene expression and modulate plant development, plant defense and stress tolerance processes (for review see Olsen et al.^13^, Nakano et al.^14^, Kim et al.^15^, Ohbayashi and Sugiyama^16^, Mathew and Agarwal^17^). NAC proteins have been documented to be involved in leaf, petal and fruit senescence in *Arabidopsis thaliana*^3,15,18–30^, *Solanum lycopersicum*^31–33^, *Oryza sativa*^34–38^, *Hordeum vulgare*^39,40^, *Glycine max*^41^, *Bambusa emeiensis*^42^, *Trifolium pratense*^43^, *Helianthus annuus*^44^, *Gossypium hirsutum*^45–47^, *Musa x paradisiaca*^48,49^, *Vitis vinifera*^50^ and *Nicotiana tabacum*^51^. Among those, several *NAC* genes are linked to a stay-green phenotype. This term is used to indicate cultivars, varieties, transgenic or knock-out lines able to maintain their green color longer than wild-type plants. In these plants, long-lasting leaf coloration is correlated to durable chlorophyll accumulation compared to wild-type plants or standard varieties, and it is often associated with delayed senescence^52^. In Arabidopsis, senescence mechanisms induce the expression of *ORESARA1* (*ORE1*) and *ORE1 SISTER1* (*ORS1*) genes. ORE1 activates program cell death and ORS1 participates to salt-induced senescence; the corresponding knock-out mutant plants display a stay-green phenotype and delayed senescence^19,20,22,26^. Conversely, the disruption of *VND-INTERACTING2* (*VNI2*) and *JUNGBRUNNEN1* (*JUB1*)—which also encode for two NAC proteins—causes early senescence while their overexpression induces a stay-green phenotype^24,53^. Recently, it was demonstrated that transgenic tomato lines, with reduced accumulation of *SlNAP2* messenger (*Solanum lycopersicum* NAC-like, activated by Apetala3/Pistillata), display a stay-green phenotype even upon ABA (abscisic acid) application^33^.

In this manuscript, we describe the role of *Solyc12g036480,* which encodes a NAC transcription factor able to modulate leaf senescence in tomato. We demonstrate that *Solyc12g036480* downregulation, achieved via Virus-induced gene silencing (VIGS), confers longer life span and delayed overall senescence in tomato plants; for this reason we named this gene *HḖBĒ* (*HEB*) after the Greek youth goddess.

## Results and discussion

### *HEB* expression analyses

The tomato NAC TFs family counts 101 members and only few of them have been functionally characterized. As yet, tomato NAC proteins have been described as involved in defense responses, stomata opening and closure, drought tolerance, flower-boundary morphogenesis, leaf senescence and fruit ripening^33,54–57^. Among these 101 NAC members, we have selected *Solyc12g036480*/*HEB* for a deeper characterization.

**Figure 1:**
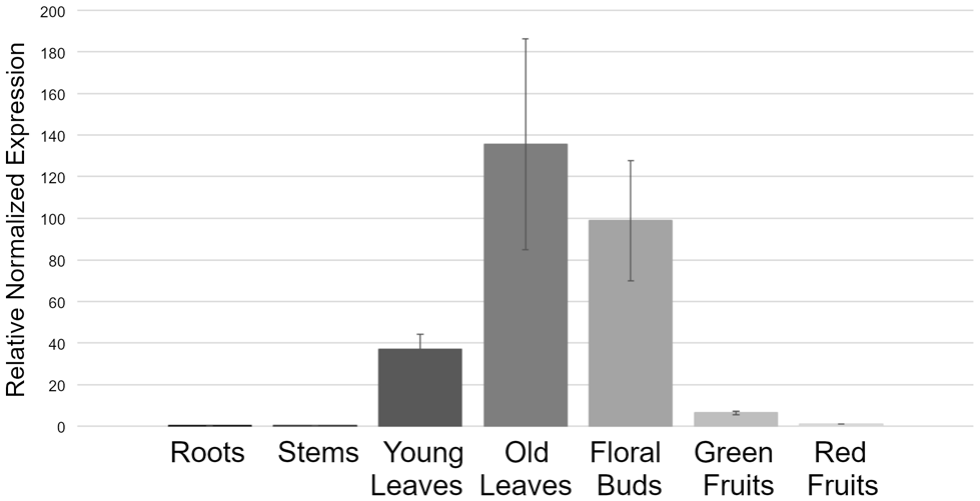
qRT-PCR performed on *HEB* transcript in different organs at different developmental stages. *HEB* is transcribed in leaves (young and senescing) and in flower buds. Bars represent the average of three technical replicates and error bars indicate standard deviation. Three independent replicates were performed and a representative experiment is shown.

According to the transcriptome data collection of the Tomato Genome Consortium, *HEB* is equally transcribed in leaves and roots, but from the experimental data of Huang and Schiefelbein, *HEB* messenger is not detected in roots^58,59^. In order to define temporally and spatially *HEB* expression pattern, quantitative Real-Time PCRs (qRT-PCRs) were performed. Expression analyses were carried out using organs dissected by Micro-tom plants; *UBIQUITIN 3* (*UBI3*) and *ELONGATION FACTOR 1α* (*EF1α*) were used as reference genes^60^. *HEB* transcript was found in young and old leaves and in young floral buds, but its mRNA is barely detected in roots, stem, mature green and red ripe fruits [developmental stages as described in^61^ (Fig. 1)].

### Identification of *HEB* putative orthologues

To identify HEB putative orthologues in other plant species, we generated a phylogenetic tree using the first 50 sequences selected using Phytozome 12 (https://phytozome.jgi.doe.gov/pz/portal.html#). Since in the Phytozome database only *Solanum lycopersicum* and *S. tuberosum* genomes are included, we additionally screened the NCBI database (https://blast.ncbi.nlm.nih.gov/Blast.cgi). The phylogenetic tree (Supplemental Fig. S1) revealed the presence of a close NAC protein of tomato (Solyc06g069100). This might be caused by a recent duplication event since two NAC transcription factors are retrieved in other Solanaceae species, but are not found in Asteraceae. *In-silico* analysis demonstrates that *Solyc06g069100* is expressed only in flower buds before anthesis^59^. To confirm *Solyc06g069100* expression pattern, we performed qRT-PCRs on the dissected Micro-tom organs previously used (Supplemental Fig. S2a). *Solyc06g069100* is highly transcribed in floral buds, but its messenger is poorly detected in old leaves and green fruits. We identified a single putative orthologue of *HEB* in species of the genus Arabidopsis (*A. halleri*, *A. lyrata* and *A. thaliana*) and Capsella (*C. grandiflora* and *C. rubella*) that belong to the Brassicaceae lineage I^62^. However, more than one putative orthologue is retrieved in lineage II^62^ (*Brassica rapa* and *Eutrema salsugineum)*. The putative orthologue in Arabidopsis (At*NAC058*), is, to date, the only one characterized: it is expressed in fruits and, its protein product participates in controlling silique maturation and senescence^63^. *HEB* and its Arabidopsis putative orthologue At*NAC058* are expressed in different tissues, nonetheless sequence identity and/or shared synteny is not sufficient to imply functional similarity. Orthologues as such are strictly the result of speciation, and in this case evolutionary convergence should also be taken into account^64^.

### *HEB* silencing through VIGS assay

To functionally characterize *HEB* we transiently silenced its expression in developing leaves by using Virus-induced gene silencing (VIGS). VIGS has been extensively employed in Solanaceae species, such as tobacco plants, that can be infected with an efficiency near 100%^65^.

VIGS technique exploits the post-transcriptional gene silencing to temporary target a selected gene (for a review see Lange et al.^66^). VIGS benefits of RNAi-mediated antiviral defense mechanisms that naturally occur in plants. dsRNAs corresponding to the target gene are produced and cleaved by the ribonuclease DICER into siRNAs oligonucleotides of 21 to 24 bp, that are used to drive the RISC complex (RNA-Induced Silencing Complex) to specifically degrade the selected transcript^67^.

The silencing of the *PHYTOENE DESATURASE* (*PDS*) gene has been used as positive control of the infiltration protocol. PDS is an enzyme necessary for carotenoid biosynthesis, therefore the successfully infected plants are recognizable for the photo-bleached leaves and fruits^68,69^. Agrobacterium-infiltrated tomato plants bear fruits that fail to accumulate lycopene and thus display an altered pigmentation^70^. We obtained the photo‐bleached phenotype in 6 out of 10 tomato plants (60% efficiency), in agreement with Liu and collaborators^69^. We were able to silence *SlPDS* in leaves, flowers and fruits (Supplemental Fig. S3).

The fragment used to silence *HEB* was identified using the SGN VIGS Tool on the Sol Genomics Network website (https://vigs.solgenomics.net/). A 499 bp target region was selected, spanning from 392–891 bp of the coding sequence, therefore excluding the 5′ region which contains the highly conserved NAC domain (Supplemental Fig. S2b). Such a fragment silences specifically *HEB* and does not affect the expression of the close NAC gene *Solyc06g069100*, as shown by the alignment in Supplemental Fig. S2b. The target region was amplified using cDNA from 15 days old tomato seedlings and cloned into pTRV2-*Gw* plasmid^71^. The silencing of *HEB* in tomato leaves was performed in two biological replicates. Plants were infected with both pTRV1 (coding for viral functions such as replication and movement) and pTRV2-*HEB* (which encodes the coat protein and the sequence of interest). Furthermore, control groups were set: plants co-infiltrated with pTRV1 and pTRV2-*GFP*, and not infected plants. As negative control, we decided to replace the gateway cassette with a reporter gene, in our case GFP (Green Fluorescent Protein) from jellyfish. Indeed the *Gw* cassette^71^ aligns with the tomato genome from nucleotide 207 to 896.

**Figure 2:**
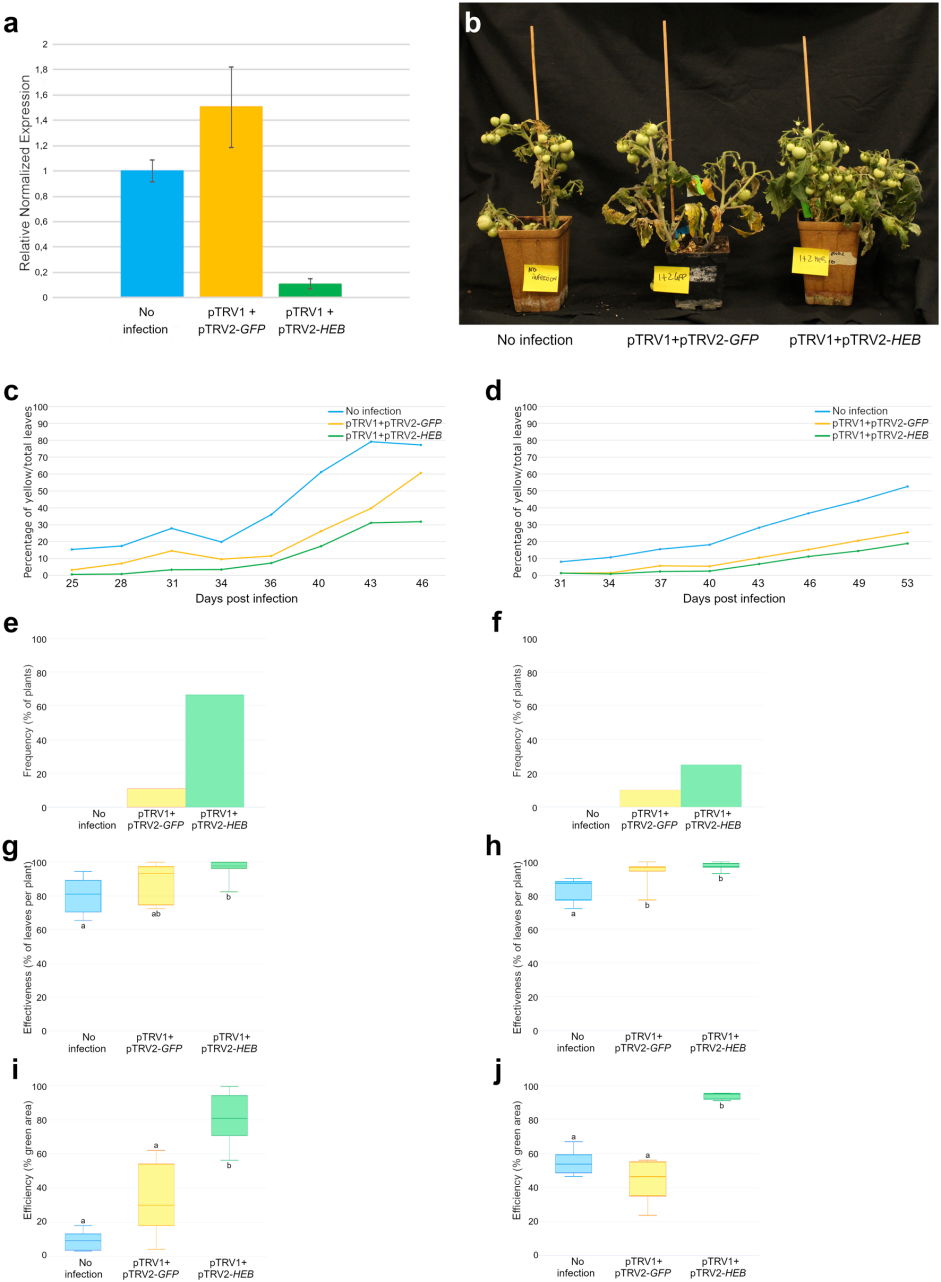
(**a**) qRT-PCR analysis to evaluate *HEB* downregulation, cDNA of terminal leaflets at 24 dpi has been used; *HEB* silencing occurs only in plants co-infiltrated with both pTRV1 and pTRV2-*HEB*. Bars represent the average of three technical replicates and error bars indicate standard deviation. Three independent replicates were performed and a representative experiment is shown. (**b**) From the left: not infected plants, pTRV1 + pTRV2-*GFP* and pTRV1 + pTRV2-*HEB*. *HEB* downregulation prevents senescence, pTRV1 + pTRV2-*HEB* co-infected plants display a stay-green phenotype. (**c**–**j**) Quantification of the phenotype and VIGS evaluation over the two infections performed: (**c**,**e**,**g**,**i**) refer to the first infection; (**d**,**f**,**h**,**j**) refer to the second infection. (**c**,**d**) Percentage of yellow leaves (number of yellow leaves/number of total leaves), data were collected by two different infections (n = 10 plants for each infection). (**e**,**f**) *HEB* silencing frequency: percentage of plants that exhibited green leaves. 10 plants were infected per each replica. (**g**,**h**) *HEB* silencing effectiveness, percentage of green leaves (number of green leaves/number of total leaves); data were collected by two independent infections [n = 310 leaves for the first infection (**g**), n = 600 leaves for the second infection (**h**)]. (**i**,**j**) *HEB* silencing efficiency: percentage of green leaf area on the total leaf area, referred to two different infections (n = 5 for each infection). Letters above or below the bars (**g**-**j**) display statistical difference based on Tukey HSD test at P ≤ 0.05.

Infiltrations were performed on young leaves at 28 days after sowing (n = 20). In the first infection, leaves were collected at 46 dpi (days post infection), in the second infection at 53 dpi since the two groups of plants germinated and grew differently. To confirm the ability of the construct to downregulate *HEB*, we extracted total RNA from leaves before the appearance of any visible phenotype, using leaves at 24 dpi. The terminal leaflet from the third node of each plant have been collected. The analysis of *HEB* expression by qRT-PCR confirmed a reduction of *HEB* transcripts only in leaves infected with pTRV1 and pTRV2-*HEB* (Fig. 2a). These data clearly indicate that, as expected, silencing of *HEB* occurred only when pTRV1 and pTRV2-*HEB* are Agrobacterium-infiltrated and *HEB* downregulation anticipates the appearance of any visible phenotype.

### Silencing of *HEB* leads to a forever-green phenotype in tomato leaves

Plants infiltrated with both pTRV1 and pTRV2*-HEB* plasmids delayed senescence compared to control plants and displayed a global aging arrest (Fig. 2b). Such phenotype is particularly striking in older plants: although control plants are approaching the end of their life cycle, the pTRV1 and pTRV2*-HEB* infected plants still have leaves with extended greenness (Supplemental Fig. S4,b). To quantify the phenotype, we calculated the percentage of senescing leaves on total leaves (Supplemental Fig. S5). The yellow leaves were rated visually, we counted the number of yellow leaves approximately every 3 days for 20 days after the appearance of the first yellow spots (Fig. 2c,d). In both the infections, thae not infected control individuals showed the highest percentage of senescing leaves, while the pTRV1 + pTRV2*-HEB* plants the lowest (Fig. 2c,d). Such a trend is maintained during the entire time frame considered.

As suggested by Broderick and collaborators^72^, we estimated the quality of the VIGS analysis calculating (i) the silencing frequency, the percentage of plants with visible silencing on total plants; (ii) the silencing effectiveness, the percentage of leaves with visible silencing on total leaves; (iii) the silencing efficiency, that is the percentage of green leaf areas on total leaf surface (Fig. 2e–j). pTRV1 + pTRV2*-HEB* co-infected plants showed the highest percentages in frequency, effectiveness and efficiency compared to control plants, meaning that the silencing occurred in a significant and stable way. On the contrary, not infected plants displayed the lowest frequency, effectiveness and efficiency. The number of green leaves of pTRV1 + pTRV2*-GFP* co-infected plants was similar to the one estimated for pTRV1 + pTRV2*-HEB* plants but, in each leaf, the green areas were smaller compared to the pTRV1 + pTRV2*-HEB* ones (Fig. 2i,j). These data indicate that *HEB* downregulation mainly delays leaf senescence thus, in the end, plant life span is prolonged.

The tomato primary shoot meristem produces 7–12 leaves, then it undergoes to the reproductive transition and turn into an inflorescence^73^. The sympodial axillary meristem develops in the axil of the last leaf giving rise to only three leaves, but again in the axil of the last leaf a new sympodial meristem develops. Although HEB prolongs plant life span, it does not affect the ability of the plant to produce new leaves; indeed the number of leaves produced by the different groups of plants did not change significantly (Supplemental Fig. S4c). Furthermore, also the number of flowers and fruits is not stricken by *HEB* downregulation (Supplemental Fig. S4d). These evidences suggest that HEB is able to control leaves life span without affecting the meristem fate.

**Figure 3:**
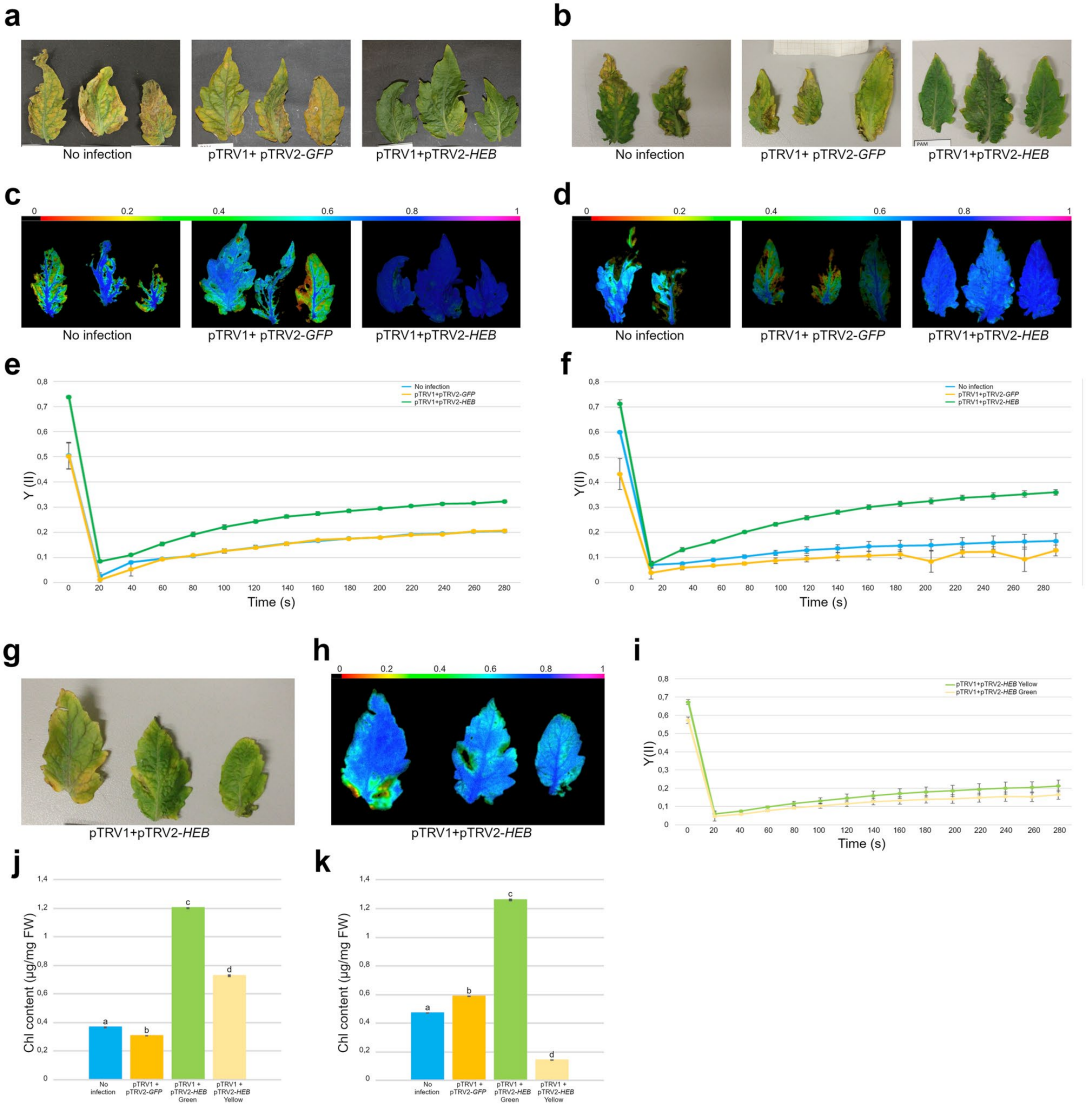
(**a**–**f,j**,**k**) Analyses of the phenotype over the two infections performed: (**a**,**c**,**e**,**j**) refer to the first infection; (**b**,**d**,**f, k**) refer to the second infection. (**a**,**b**) Comparison between pTRV1 + pTRV2*-HEB* infected leaves and controls (No infection and pTRV1 + pTRV2*-GFP*) revealed that *HEB* silencing delays senescence. (**c**,**d**) Visual aspect of PSII quantum yields (*F*_*v*_*/F*_*m*_, Imaging PAM) of pTRV1 + pTRV2*-HEB* infected leaves and controls. The tissue color indicates the maximum quantum yield of PSII, ranging from black (no efficiency) to violet (maximum efficiency) as shown by the colored bar on the top. (**e**,**f**) Y(II) of pTRV1 + pTRV2*-HEB* infected leaves and controls. Dots represent the average of 3 technical replicates and error bars indicate standard error. In each time point, statistical differences between pTRV1 + pTRV2*-HEB* and the controls was assessed with Tukey HSD test at P ≤ 0.05. A representative result from three independent experiments is shown. (**g**) pTRV1 + pTRV2*-HEB* older leaves (65 dpi) display yellow and light green spots. (**h**) Visual aspect and PSII quantum yield (*F*_*v*_*/F*_*m*_, Imaging PAM) of pTRV1 + pTRV2*-HEB* older leaves. (**i**) Measurement of Y(II) in pTRV1 + pTRV2*-HEB* older leaves. Dots represent the average of three technical replicates and error bars indicate standard error. In each time point, statistical differences between pTRV1 + pTRV2*-HEB* green and yellow sectors was assessed with Tukey HSD test at P ≤ 0.05. A representative result from three independent experiments is shown. (**j**,**k**) Chlorophyll content quantification in green/yellow sectors of pTRV1 + pTRV2*-HEB* infected leaves and in controls at 53 (**j**) and 60 (**k**) dpi. Bars represent the average of 3 technical replicates and error bars indicate standard error. Letters above the bars (**j**,**k**) display statistical difference based on Tukey HSD test at P ≤ 0.01. A representative result from three independent experiments is shown.

To better characterize the effects of *HEB* downregulation, we measured the maximum quantum yield (*F*_*v*_*/F*_*m*_^74^) of the PSII (Photosystem II) as indicator of photosystem integrity. For each group of plants, we analyzed three biological replicates per each infection. Visual comparisons of the leaves already indicated that the silencing of *HEB* delays leaf senescence (Fig. 3a,b), these observations were further confirmed by the IMAGING PAM fluorometer (Fig. 3c,d).

As expected, pTRV1 + pTRV2*-HEB* showed the highest effective quantum yield (Y(II)) after 280 s of actinic light exposition (Fig. 3e,f). pTRV1 + pTRV2*-HEB* retained an optimal photosynthetic capacity, like younger leaves, in a statistically significant way in all the considered time points.

### *HEB* controls senescence in tomato leaves

**Figure 4:**
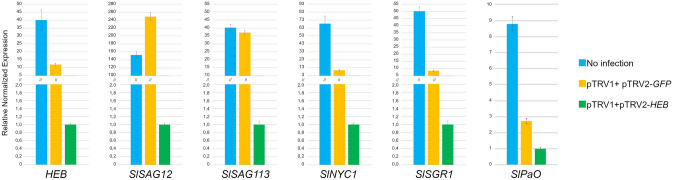
Quantification by qRT-PCR of the expression of *HEB*, *SlSAG12*, *SlSAG113*, *SlNYC1*, *SlSGR1* and *SlPaO* from pTRV1 + pTRV2-*HEB*, pTRV1 + pTRV2*-GFP* and not infected leaves at the same developmental stage (65 dpi). *HEB* and all the senescence-related genes resulted to be downregulated in pTRV1 + pTRV2-*HEB* leaves compared to the controls. Bars represent the average of three technical replicates and error bars indicate standard deviation. Two independent replicates were performed and a representative experiment is shown.

Leaf senescence is a progressive process; in adult leaves, yellowing first appears in discrete spots which progressively enlarge. In pTRV1 + pTRV2-*HEB* leaves previously analyzed (Fig. 3a,b), yellowing was not present differently from the controls. In order to evaluate how the silencing of *HEB* affects senescence, we sampled older leaves, at 65 dpi, when yellow spots appeared also in pTRV1 + pTRV2-*HEB* leaves. Total RNA was extracted from pTRV1 + pTRV2-*HEB*, pTRV1 + pTRV2*-GFP* and not infected leaves. Quantification of *HEB* transcripts by qRT-PCR confirmed a downregulation in pTRV1 + pTRV2-*HEB* leaves compared to pTRV1 + pTRV2*-GFP* and not infected leaves (Fig. 4).

To understand how *HEB* expression impacts leaf senescence, we quantified the transcript accumulation of a number of genes known to be involved in this process. In particular, we measured the relative expression of two *Solanum lycopersicum SENESCENCE ASSOCIATED GENES* (*SlSAGs*)—*SlSAG12* (*Solyc02g076910*) and *SlSAG113* (*Solyc05g052980*)—as well as three other genes involved in chlorophyll degradation, *NON-YELLOW COLORING 1* (*SlNYC1, Solyc07g024000*), *PHEOPHORBIDE a OXYGENASE* (*SlPaO, Solyc11g066440*) and *STAY-GREEN 1* (*SlSGR1*, *Solyc08g080090*).

*SAG12* encodes a cysteine protease and it has been widely used as senescence-associated reference gene; in Arabidopsis it is abundant in senescent leaves^75^, when the yellowing is clearly visible^76^. SAG12 protein localizes in the senescence-associated vacuoles and participates to RuBisCO degradation^77,78^. However, the Arabidopsis *sag12* mutant does not show any phenotype and leaf senescence progression appears normal^79^. Conversely, two homologs of *SAG12* in rice (*OsSAG1-2* and *OsSAG2-2*) negatively regulate senescence-related cell death^80^. In tomato, *SlSAG12* has been extensively used as senescence-associated reference gen^33,81,82^. The second candidate gene, *SAG113*, encodes a phosphatase 2C expressed in ageing tissues and has already been reported as a senescence marker^32,33^. In Arabidopsis, SAG113 is a negative regulator of stomatal movement, its disruption causes tissue dehydration followed by senescence, and *sag113* mutant shows delayed leaf senescence^23,83^.

qRT-PCR results showed downregulation of *SlSAG12* and *SlSAG113* in pTRV1 + pTRV2-*HEB* leaves (Fig. 4) compared to the controls, thus suggesting a delay in the onset of ageing.

During fruit ripening and the establishment of leaf senescence, Chls are massively degraded by plastid proteins. In higher plants, Chl degradation begins with the reduction of Chl*b* to Chl*a*, mediated by Chl*b* reductase and 7-hydroxymethyl-chlorophyll *a* reductase^84,85^. Following this event, Chl degradation occurs in two steps, firstly, the pigments are converted into a colourless, blue-fluorescing product named primary fluorescent Chl catabolites (*p*FCC). This step is catalysed by chlorophyllase (Chlase), Mg-dechelatase, pheophorbide a oxygenase and red chlorophyll catabolite reductase. Afterwards, *p*FCCs are modified and exported into the vacuole, leading to their non-enzymatic isomerization into non-fluorescent chlorophyll catabolites, called NCCs^86^.

To determine whether the Chl breakdown is affected by *HEB* downregulation, we selected three different genes involved in different stages of Chl breakdown. *SlNYC1* encodes the Chl*b* reductase which converts Chl*b* to Chl*a*^84^, while *SlPaO* codes for a pheophorbide *a* oxygenase that cleaves the porphyrin ring of Pheide *a,* producing oxidized red Chl catabolite. PaO enzymatic activity participates as well in the de-greening process^87–90^. Finally, *SlSGR1* was selected as it is implicated in the regulation of all the above-mentioned genes, via translational or post translational regulation^84,91,92^.

The data generated by our expression quantitation study (Fig. 4) suggest that *HEB* downregulation prevents the activation of chlorophyll degradation pathways. Indeed, in pTRV1 + pTRV2-*HEB* leaves, *SlNYC1*, *SlSGR1* and *SlPaO* transcripts are reduced compared to control leaves, implying a more efficient photosynthetic performance due to chlorophyll integrity.

Taken together, these data suggest that *HEB* is a positive regulator of senescence mechanisms in tomato leaves, since *HEB* transient silencing in leaves fails to activate the correct aging pathways, leading to a stay-green phenotype.

### *HEB* downregulation defers the ageing program

To better evaluate HEB involvement in senescence progression, we measured the maximum and effective quantum yield and the chlorophyll (Chl) content of yellow and green sectors of *HEB* silenced leaves and control leaves at 65 dpi. As expected from the visual output of the IMAGING PAM fluorometer (Fig. 3g,h), the yellow regions of the leaves displayed a reduced photosynthetic efficiency (Fig. 3i). In particular, the maximum quantum yield is statistically significant comparing yellow and green portions. These observations were also confirmed by the Chl quantification (Fig. 3j,k) since the green regions of pTRV1 + pTRV2-*HEB* leaves contain markedly higher Chl amount compared to the yellow regions and to the controls.

Many NAC transcription factors are known to be involved in the control of leaves senescence in tomato, such as *SlNAP2*, *NOR* (*NON-RIPENING*) and *SlORE1S02*^31–33^. Interestingly, *slnap2* knockdown mutants and *nor* mutants display a delayed senescence only when excised leaves undergo dark induced senescence, however the physiological senescence is comparable to the wild-type plants. Moreover, in *nor* mutants or *NOR* overexpressing lines, Chl content is similar to the control plants, when grown in light, and differences appear only after 14 days of continuous darkness^32^. A similar phenotype has been described for *slnap2* mutant and *SlNAP2* overexpressing lines: a difference in Chl content compared to the control is measurable only after 14 days of darkness^33^. In our experiments, leaves were not subjected to dark adaptation before measurement of Chl content, but rather exposed to canonical long day conditions (16 h light/8 h dark). This suggests that HEB positively modulates leaf senescence and is a strong promoter of the physiologic aging programs.

**Figure 5:**
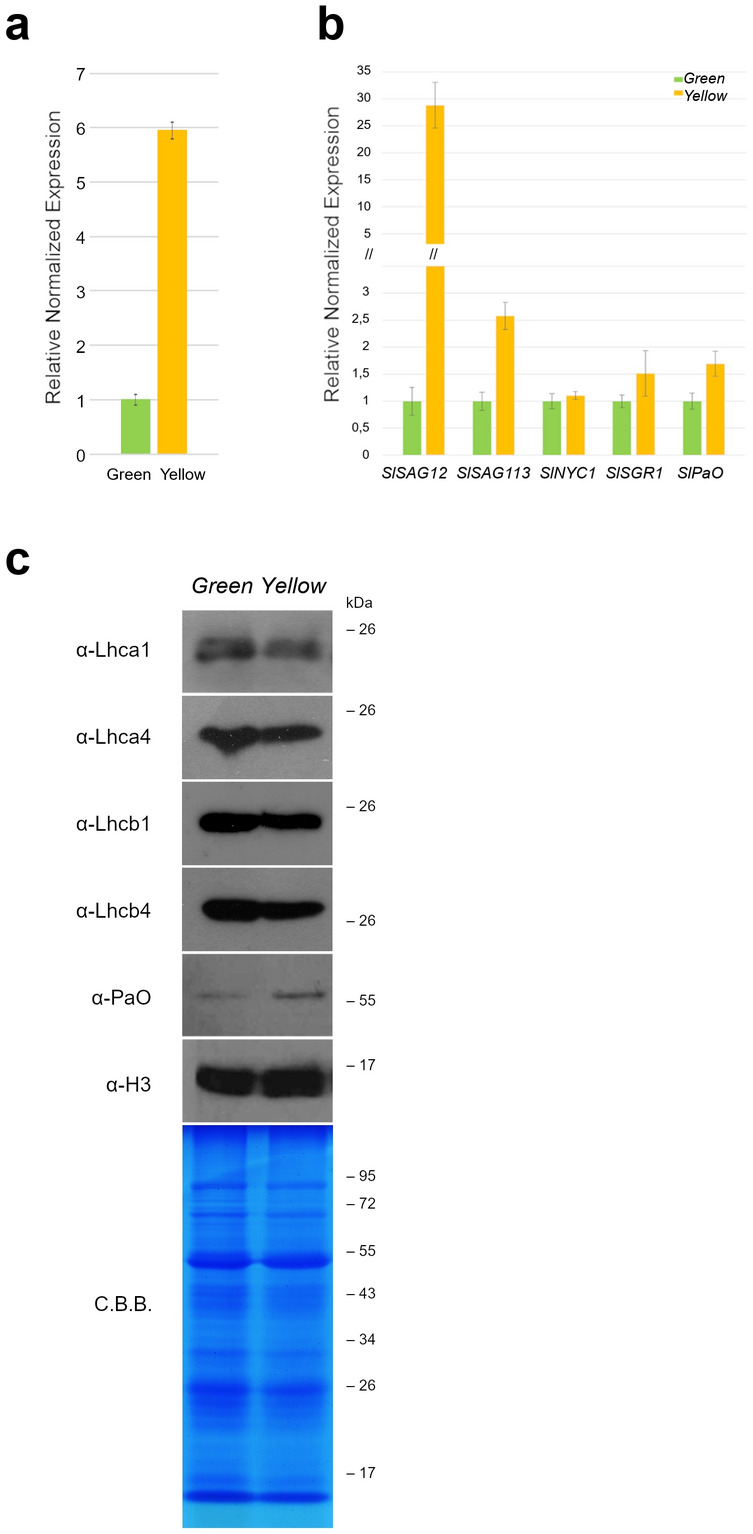
(**a**) qRT-PCR analysis on mRNAs from green and yellow portions of pTRV1 + pTRV2-*HEB* infected leaves. The expression of *HEB* is strongly reduced in the green sections. Bars represent the average of three technical replicates and error bars indicate standard deviation. Three independent replicates were performed and a representative experiment is shown. (**b**) Quantification by qRT-PCR of the expression of senescence-related genes *SlSAG12*, *SlSAG113*, *SlNYC1*, *SlSGR1* and *SlPaO*, in green/yellow regions of pTRV1 + pTRV2-*HEB* infected leaves. A general downregulation of senescence-associated genes is recorded in the green sectors, especially *SlSAG12* is strongly reduced. Bars represent the average of three technical replicates and error bars indicate standard deviation. Three independent replicates were performed and a representative experiment is shown. (**c**) Immunoblot analyses were performed to evaluate the presence and the amount of chlorophyll associated proteins in green/yellow regions. While the chlorophyll-binding proteins Lhca1, Lhca4, Lhcb1 and Lhcb4 accumulation is slightly reduced in the yellow sectors, compared to the green ones, the catalytic enzyme PaO, involved in chlorophyll degradation, is more abundant in the yellow areas. The histone protein H3 was used as loading control, together with a Coomassie Brilliant Blue (C.B.B.) staining of the SDS-PAGE. A representative result from three independent experiments is shown.

We also evaluated the expression of *SlSAG12*, *SlSAG113*, *SlNYC1*, *SlSGR1* and *SlPaO* in the different areas of the same pTRV1 + pTRV2-*HEB* leaf. Total RNA was extracted from three independent replicates, carefully separating dark and pale regions of the leaves, and first used to quantify *HEB* transcript accumulation. A strong downregulation of *HEB* was detected in the dark portions of the leaves (Fig. 5a). The senescence-associated genes and the chlorophyll-related genes transcripts were then quantified by qRT-PCR, results showed downregulation of all the targets in the green sectors of the leaves (Fig. 5b).

To clarify whether *HEB* silencing can also impact SlPaO protein accumulation, we performed an immunoblot analyses. The protein HISTONE 3 (H3) was used as control (Fig. 5c). In our analysis, PaO poorly accumulated in the green sectors, while in the senescing yellow sectors was more abundant. To corroborate the role of HEB, and to establish the level of accumulation of photosystems I and II, we investigated the accumulation of the chlorophyll binding proteins of the light-harvesting complexes of Photosystem I and II. We detected a decreased level of Lhca1, Lhca4, Lhcb1 and Lhcb4 proteins in the yellow sectors of the leaves. This analysis indirectly supports the hypothesis that *HEB* is necessary to trigger the senescence process in leaf: indeed with the Lhca1, Lhca4, Lhcb1 and Lhcb4 antibodies we detected a reduced level of antenna proteins, directly associated to Chl. These data corroborate the findings that the silencing of *HEB* in green leaf sectors led to a marked delay in the ageing program, preventing the accumulation of PaO protein, thus maintaining high level of Lhc proteins.

### Can HEB bind *PaO* promoter?

It was previously described that SlNAP2 controls organ ageing directly binding the regulative regions of *SlSGR1* and *SlPaO*^33^. Similarly, NOR is able to directly bind the promoter of *SlSAG113* and *SlSGR1* but not *SlPaO*^32^. According to our data, HEB mechanism is comparable to NOR and SlNAP2 ones, since its expression positively correlates with the expression of such genes. As of yet, due to a lack of structural characterization of HEB binding site, we cannot predict whether these differently regulated genes are direct target of HEB. In-silico research of the promoter region of *SlSAG12, SlSAG113*, *SlNYC1, SlSGR1* and *SlPaO* returned putative binding sites for several NAC genes. We detected a putative binding site for AtNAC058 on the promoter region of *SlSAG113*, *SlSGR1*, *SlPaO* and *SlNYC1* (Supplemental Fig. S6). To verify the ability of HEB to bind the regulative regions of *SlPaO*, we used a yeast 1-Hybrid assay (Supplemental Fig. S7). HEB was fused with the GAL4-AD (Activation Domain) whilst a fragment of 1.45 kb that include the 5′ region of *SlPaO* was cloned into pHis2 vector (see Materials and methods). Both constructs were introduced into a diploid yeast via mating and colonies were selected on a medium lacking histidine to assay protein–DNA interaction. No clear yeast growth was observable, implying that no interaction occurs among HEB and *SlPaO* promoter.

### *HEB* overexpression doesn’t lead to transcript accumulation

In order to confirm that HEB is a senescence positive regulator, we generated transient gain of function plants. We used Agrobacterium to infect tomato leaves with a construct bearing 35S::*HEB* (n = 2). Each leaf was ideally divided in two parts, separated by the midrib; one half was infected with 35S::*HEB* construct while the other half with a control construct bearing the 35S::*GUS* (β-glucuronidase) reporter (Supplemental Fig. S8a). 10 days after the infection leaves were collected (Supplemental Fig. S8b) and the IMAGING PAM fluorometer was used to evaluate the photosynthetic efficiency of the two halves of the leaves (Supplemental Fig. S8c). The portions of the leaves infected with the 35S::*HEB* construct revealed a slight increase in the photosynthetic yield compared to the control region (Supplemental Fig. S8d). In order to evaluate the expression level of β-glucuronidase and *HEB*, the total RNA was extracted from the two halves of the leaves. qRT-PCRs revealed that the β-glucuronidase gene was overexpressed in the 35S::*GUS* half of the leaves (Supplemental Fig. S8e). A small amount of β-glucuronidase was also detected in the 35S::*HEB* half of the leaves, and this might be a collateral effect of the procedure. Conversely, *HEB* was not overexpressed in both the leaves portions (Supplemental Fig. S8e), this suggests that HEB transcript is difficult to accumulate and might be quite unstable thus preventing the possibility to run analysis that request transient over- or mis-expression.

### The fountain of youth: possible application of a forever young plant

One of the main goals of crop science is the improvement of traits that can increase plant yield and biomass. This can be achieved by several ways, one of which is the extension of the photosynthetic ability by prolonging a plant’s lifecycle. Keeping a plant in a stay-green status can be reached in different manners, known as “the five ways to stay green”: (i) delaying the initiation of leaf senescence, (ii) slowing down the rate of leaf senescence, (iii) delaying chlorophyll degradation, (iv) causing tissue death (for example by freezing, boiling or drying) or (v) enhancing greenness^93^. HEB falls in the first category of stay-green phenotype, since it causes a delay in the initiation of leaf senescence, and here we have demonstrated that *HEB* silencing defers ageing progression leading to a stay-green phenotype and prevents the transcription of genes involved in chlorophyll degradation (Figs. 2, 3, 4, 5).

Interestingly, the longer life span of leaves does not affect flower and fruit yield in the time frame considered (20 days, Supplemental Fig. S4). In cereals, it was shown that the stay-green phenotype has a negative effect on yield since prolonged lifecycle of crops causes nutrient dilution. This phenomenon is known as the “dilution effect”, when the relationship between crop yield and mineral concentrations become unfavorable^94^. For instance, silencing of the wheat NAC gene *NAM-B1* delays senescence, though concurrently, protein content and presence of elements such as zinc and iron in seeds are dramatically compromised^95^. In tomato *nor* mutants, fruit ripening and dark-induced senescence are delayed^32^, albeit at the cost of fruit quality: ethylene and pigment biosynthesis are both downregulated, while cellulose synthase proteins are upregulated, causing an increase of fruit firmness. Nutrient biosynthesis was also found to be impaired, however resistance to pathogens appears enhanced^96^. Considering these findings, the study of *HEB* fruits to determine potential impacts of prolonged lifecycle on nutrient biosynthesis and/or pathogen resistance is an extremely compelling future endeavor.

## Conclusion

In this work we silenced the putative tomato NAC transcription factor *Solyc12g03648*/*HEB* through VIGS assay. The results we uncovered suggest that *HEB* is involved in the regulation of leaf senescence in tomato, acting as a positive regulator. Reduction of *HEB* transcript leads to the fail-activation of *SENESCENCE ASSOCIATED GENES* (*SAGs*) and lack of chlorophyll degradation mechanisms, ultimately delaying leaf senescence and prolonging the life span of the entire plant. Further research will be focused on identifying the molecular mechanisms through which *HEB* controls the ageing processes, by identifying its target genes and interactors. With this research we identified a tomato gene involved in delaying leaf senescence, laying the base for future applications which will allow the cultivation of longer-lived crops.

## Material and methods

### Plant material and growth conditions

Micro-Tom tomato plants were grown on soil under greenhouse condition with a 16 h light/8 h dark cycle at 22/18 °C. For VIGS assay we Agrobacterium-infiltrated young leaves 28 days after sowing.

### Phylogenetic analysis

To identify *HEB* putative orthologues, we screened the Phytozome database (https://phytozome.jgi.doe.gov/pz/portal.html) using HEB protein sequence to conduct blastp analyses; the first 50 sequences obtained were then selected for the phylogenetic tree. For the Solanaceae sequences not present in the Phytozome database we screened the NCBI database (https://blast.ncbi.nlm.nih.gov/Blast.cgi) using HEB protein sequence to conduct blastp.

We also queried blastp on the Phytozome database using HEB protein sequence against *Solanum lycopersicum* proteome and AtNAC058 protein against *Arabidopsis thaliana* proteome. We selected the proteins with the highest score, and we used them as control of the phylogenetic tree. All the selected proteins were aligned with MUSCLE. The phylogenetic tree was constructed with MEGAX (https://www.megasoftware.net/) using a Maximum Likelihood method (JTT protein model, bootstrapping of 100).

### RNA extraction, cDNA synthesis and expression analysis

Total tomato RNA was extracted using the cetyl trimethyl ammonium bromide (CTAB) protocol (adapted from Chang et al.^97^). Genomic DNA was removed using TURBO™ DNase (Invitrogen™) according to the manufacturer’s instructions. The RNA was reverse transcribed using the iScript™ gDNA Clear cDNA Synthesis Kit (Biorad) and the cDNA was used as template in qRT-PCR reactions. qRT-PCR was carried out on a CFX96 Real-Time system (Bio-Rad), using the primer pairs reported in Supplemental Table S1. The *UBI3* and *EF1α* transcripts were used as internal references^60^. The Bio-Rad CFX Manager software (V3.1) was used to analyze data from three biological and three technical replicates (except where otherwise specified, see Fig. 4).

### VIGS assays

In this work we used the pTRV plasmids previously described by Orzaez et al.^71^. *HEB* and *GFP* fragments were cloned from seedling cDNA and pGREENII plasmid respectively, using the primers reported in the Supplemental Table S1. The pTRV2-*PDS* plasmid was kindly provided by Concha Gómez Mena (Instituto de Biología Molecular y Celular de Plantas, Valencia, Spain).

GV3101 Agrobacterium culture was transformed with pTRV1, containing the viral genes for replication and movement, and a second culture with pTRV2, containing the fragment for *PDS* or *HEB* or the *GFP* fragment. These cultures were used to infiltrate tomato plants (n = 10 for each construct in each infection), while plants without plasmids were used as mock (n = 10 in each infection). For the *PDS* silencing we Agrobacterium-injected 10 plants.

The infiltration was performed as described in^98^. Young leaves were infiltrated using syringes without needles, while for fruit infection we infiltrated the pedicels of the flower with a needles syringe. Tomato not infiltrated or infiltrated with pTRV1 and pTRV2-*GFP* was used as control. Each inoculation was carried out two times.

### Chlorophyll content and chlorophyll fluorescence analysis

Pigments were extracted using 90% (v/v) acetone from different portion of leaves. The chlorophyll a and b contents were measured using a spectrophotometer (Amersham Biosciences) at 663- and 645-nm wavelength. Total chlorophyll (a + b) values were determined as described previously by Arnon^99^ and normalized relative to tissue fresh weight. The pulse-modulated fluorometer IMAGING-PAM M-Series (Walz) was used to measure in vivo chlorophyll *a* fluorescence of tomato leaves^100^. Infected or not infected leaves were placed under the fluorometer and three measurements for each phenotype/infection were performed, and three biological replicates were used. Samples were first dark adapted and the fluorescence was measured as reported in^63^.

### Immunoblot analyses

For immunoblot analyses, tomato leaves were collected from plants infected with both *pTRV1* and *pTRV2- HEB*, at 46 dpi—first infection- and 53 dpi—second infection. Total protein content was extracted according to^101^. Protein extracts, corresponding to 5 mg of leaves fresh weight, were fractionated by SDS-PAGE gel (12% [w/v] acrylamide^102^) and then transferred to polyvinylidene difluoride membranes^103^. Replicate filters were cropped and immunodecorated with antibodies specific for proteins with different molecular weights, Lhca1, Lhca4, Lhcb1, Lhcb4, PaO and Histone H3. Lhca1, Lhca4, Lhcb1, Lhcb4, and PaO antibodies were obtained from Agrisera, Histone H3 antibody from Sigma-Aldrich.

### Yeast 1-hybrid assay

The *SlPaO* promoter region (*Solyc11g066440*) of 1,434 bp was amplified using a primer pair containing *Eco*RI restriction sites (Supplemental Table S1), cloned into pHis2 vector (Clontech) previously linearised using *Eco*RI. The bait plasmid (p*PaO*-pHis2) was used to transform *Saccharomyces cerevisiae* Y187 strain (Clontech). The *Solyc12g03648*/*HEB* gene (981 bp) cloned into pBlueScript II SK(+) vector was purchased from Biomatik Corporation (Cambridge, Canada), excised with *Eco*RI and *Xho*I and ligated into pGADT7 (Clontech) *Eco*RI/*Xho*I digested. The prey plasmid (*HEB*-pGADT7) was introduced into *Saccharomyces cerevisiae* AH109 strain (Clontech) and transformants mated with Y187 strain containing the bait plasmid as described by Resentini et al.^104^. Diploids were selected on medium lacking Trp and Leu. Growth diploid colonies were scraped on selective media lacking Trp, Leu and His and supplemented with 0, 1, 2 or 5 mM 3-AT (Sigma-Aldrich). Yeast 1-Hybrid was also performed using as bait and prey plasmids pHis2 and pGADT7 respectively as controls.

### Transient expression in tomato leaves

We transformed Agrobacterium strain GV3101 with 35S::*HEB* and 35S::*GUS* constructs. These cultures were used to Agrobacterium-infiltrate tomato leaves (n = 2). Briefly, Agrobacterium cultures of 35S::*HEB* and 35S::*GUS* were pre-inoculated in 5 ml liquid medium and let grow for 20 h in stirring conditions at 28 °C. OD at 600 nm was then measured, cultures were concentrated and resuspended in infiltration buffer (MgCl_2_ 10 mM, MES pH 5, 6, 10 mM, acetosyringone 150 µM) to the final OD. Cultures were grown for two hours at room temperature in stirring conditions. Each leaf was ideally divided in two halves and infiltrated with 2 ml of 35S::*GUS* suspension in one half and with 2 ml of 35S::*HEB* suspension in the other half.

## Supplementary information

Supplementary file1 (PDF 1626 kb)
