# Supplementary material for: HEBE, a novel positive regulator of senescence in Solanum lycopersicum
Source: Sci Rep. 2020 Jul 3;10:11021. doi: 10.1038/s41598-020-67937-z (PMC7335192; doi:10.1038/s41598-020-67937-z)
Supplement: Supplementary file 1 — Supplementary file1 (PDF 1626 kb) [file 41598_2020_67937_MOESM1_ESM.pdf]

Scientific Reports

HEBE, a novel positive regulator of senescence in *Solanum lycopersicum*.

Sara Forlani[§], Carolina Cozzi[§], Stefano Rosa, Luca Tadini, Simona Masiero* and Chiara Mizzotti*

Department of Biosciences, Università degli Studi di Milano, via Celoria 26, 20133 Milan, Italy

§: These authors contributed equally to this work

***Corresponding authors:**

Chiara Mizzotti, E-mail: chiara.mizzotti@unimi.it

Simona Masiero, E-mail: simona.masiero@unimi.it;

SUPPLEMENTAL FIGURES

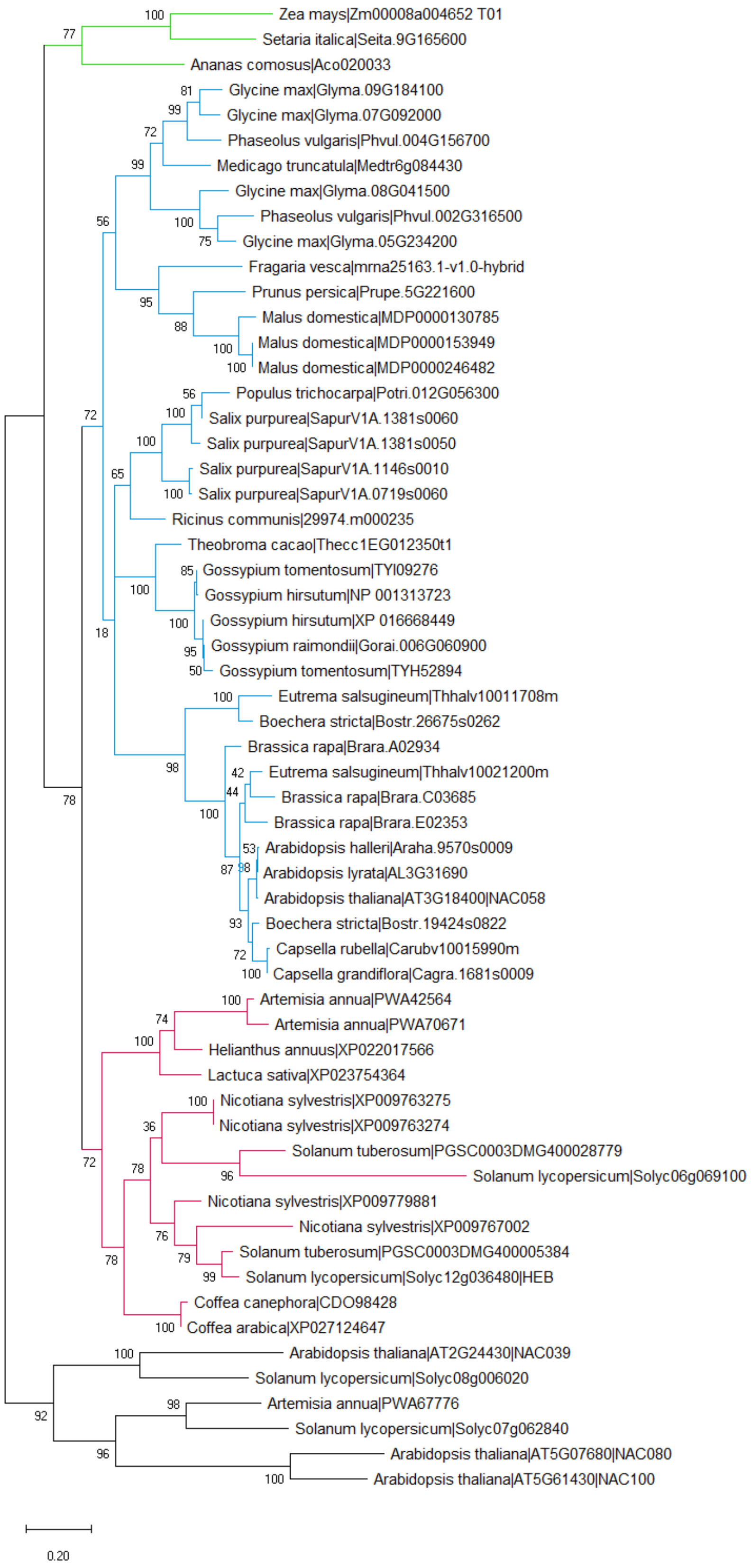

Figure S1. Phylogenetic tree of HEB putative orthologues. Putative orthologues have been identified using Phytozome and NCBI databases as reported in the material and methods section. The closest proteins from tomato and Arabidopsis was used as control and are reported in black. The monocots branch is highlighted in green, the dicots branch is divided into rosids (blue branch) and asterids (red branch).

a

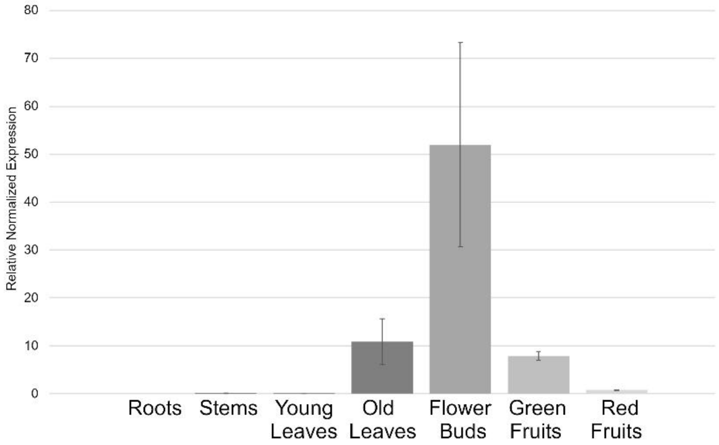

b

```
                                                       NAC domain
NAC058               ---------MEENLPPGFRFHPTDEELITHYLCRKVSDIGFTGKAVVDVDLNKCEPWDLP 51
HEB                  ---------MDENLPPGFRFHPTDEELITCYLNNKISDFNFTTRAIADVDLNKSEPWDLP 51
Solyc06g069100.1.1   MMIEQYNIMDEKNLPPGFRFHPSDEELITYYLSNKVSDFSFTTRAIADVDLNKSEPWDLP 60
                               ::**********:****** ** .*:**:.** :*:.******.******

                                                NAC domain
NAC058               AKASMGEKEWYFFSQRDRKYPTGLRTNRATEAGYWKTTGKDKEIYRSG--VLVGMKKTLV 109
HEB                  AKASMGEKEWYFFSLKDRKYPTGLRTNRATEAGYWKTTGKDKEIYRGGTGVLVGMKKTLV 111
Solyc06g069100.1.1   AKSSMGEKEWYFFSQKDRKYPTGLRSNRATEAGYWKTTGKDKEIFRGGVE-LVGMKKTLV 119
                     **:*********** :*********:******************:*.*   *********

                          NAC domain
NAC058               FYKGRAPKGEKSNWVMHEYRLESKQPFNPTNKEEWVVCRVFEKSTAAKKAQEQQPQSSQP 169
HEB                  FYRGRAPKGEKTNWVMHEYRIETTFGYKPS-KEEWVVCRVFQKSSTVKKPQPTS--SSPL 168
Solyc06g069100.1.1   FYKGRAPKGEKTNWVMHEYRLQSNLGFKPP-KEEWVVCRLFHKNSTVKKSNATS--SQPS 176
                     **:********:********:::.  ::*  ********:*.*.::.** :  .  *.
                                                   VIGS fragment

NAC058               SFGSPCDANSSMANEFEDIDELPNLNSNS---STIDYNNHIHQYSQRNVYSEDNTTSTAG 226
HEB                  SLESPCDTNYTITNELGDIELPFNFNYLTTTPSTAINNISLHNYNND-----NINLA--- 220
Solyc06g069100.1.1   DQVESISDAYTLP----------NFNIPAPHEGQIVNDISLHNYYTS-----QENMN--- 218
                     .  .  .   ::             *:*  :    .    :  :*:*           : .
                                           VIGS fragment

NAC058               LNMNMNMASTNLQSWTTSLLGPPLSPINSLLLKAFQIRNSYSFPKEMI-PSFNHSSL--- 282
HEB                  -AATREANSHPLLPWSSNLLSSNLSSVNSLLFRALQLKSYSPREQA------TTTHD--- 270
Solyc06g069100.1.1   -IVNLD-------PLPSL-----------LLYKAIQCRGNYNHQPMNIDPSINTMNHAQF 259
                        . :          :           ** :*:* :.                   .
                                           VIGS fragment

NAC058               ------QQGVSNM-------------------------------------IQNASSSS 297
HEB                  YAFMLPQENII--------------------TTQFGNDF--------AVNNIGAPSSST 301
Solyc06g069100.1.1   YHHTNSNQAIVNNNNNNNLSSPLKINKASHHIKKSLSNSLPSSSSSSSLYNTVAATTSTT 319
                           :: :                                        :    :*::
                                             VIGS fragment

NAC058               ----QVQP---QPQEEAFNM-DSIW----------------------------------- 314
HEB                  VLDNSVQQQQQQQQEQSYKLDSNIW*---------------------------------- 326
Solyc06g069100.1.1   IIPHQ-QQQQQRQPVIIYTHSPKVIHTHPRDFKALVQKLTGLSPEEVSSSPQSMPMPQYH 378
                          . *    :      :.    .:

NAC058               ------------------------------------------------------------ 314
HEB                  ------------------------------------------------------------ 326
Solyc06g069100.1.1   PKSEPINGEMDHDHDTIIGAESCFGDEQNNYHRNNMTNINTNINEKCDLLMNDDNDSVVT 438

NAC058               ------------------------------------------------------------ 314
HEB                  ------------------------------------------------------------ 326
Solyc06g069100.1.1   DENNEFNGSSCIGDNNSSASCYVPHQQLSNYTDSLFSMPSMRNSFSSSSSESIKELPDF* 497
```

Figure S2. (**a**) qRT-PCR performed on *Solyc06g069100* transcript in different organs at different developmental stages. *Solyc06g069100* is transcribed in flower buds and it is also present at low level in senescing leaves and green fruits. Bars represent the average of three technical replicates and error bars indicate standard deviation. Three independent replicates were performed and a representative experiment is shown. (**b**) In order to specifically target *HEB* transcript through VIGS, a fragment of 499 bp was selected and cloned (highlighted in red). The alignment of HEB with AtNAC058 and Solyc06g069100 protein sequence pointed out the NAC conserved domain, which was excluded to avoid off targets.

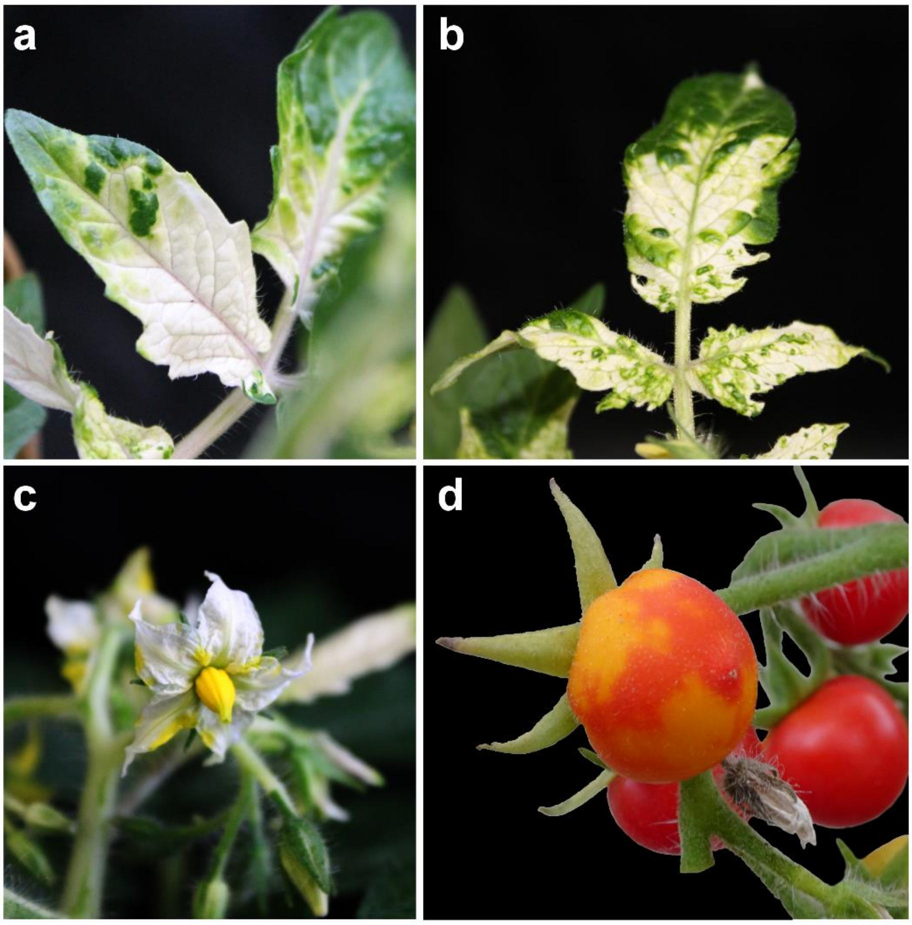

Figure S3. Silencing of the *PHYTOENE DESATURASE* (*PDS*) gene was used as control of the VIGS assay. PDS is an enzyme that participates to the carotenoid biosynthesis therefore its downregulation causes the photo-bleaching of leaves (**a**, **b**) flowers (**c**) as well as the formation of lycopene-depleted fruits with an altered coloration (**d**).

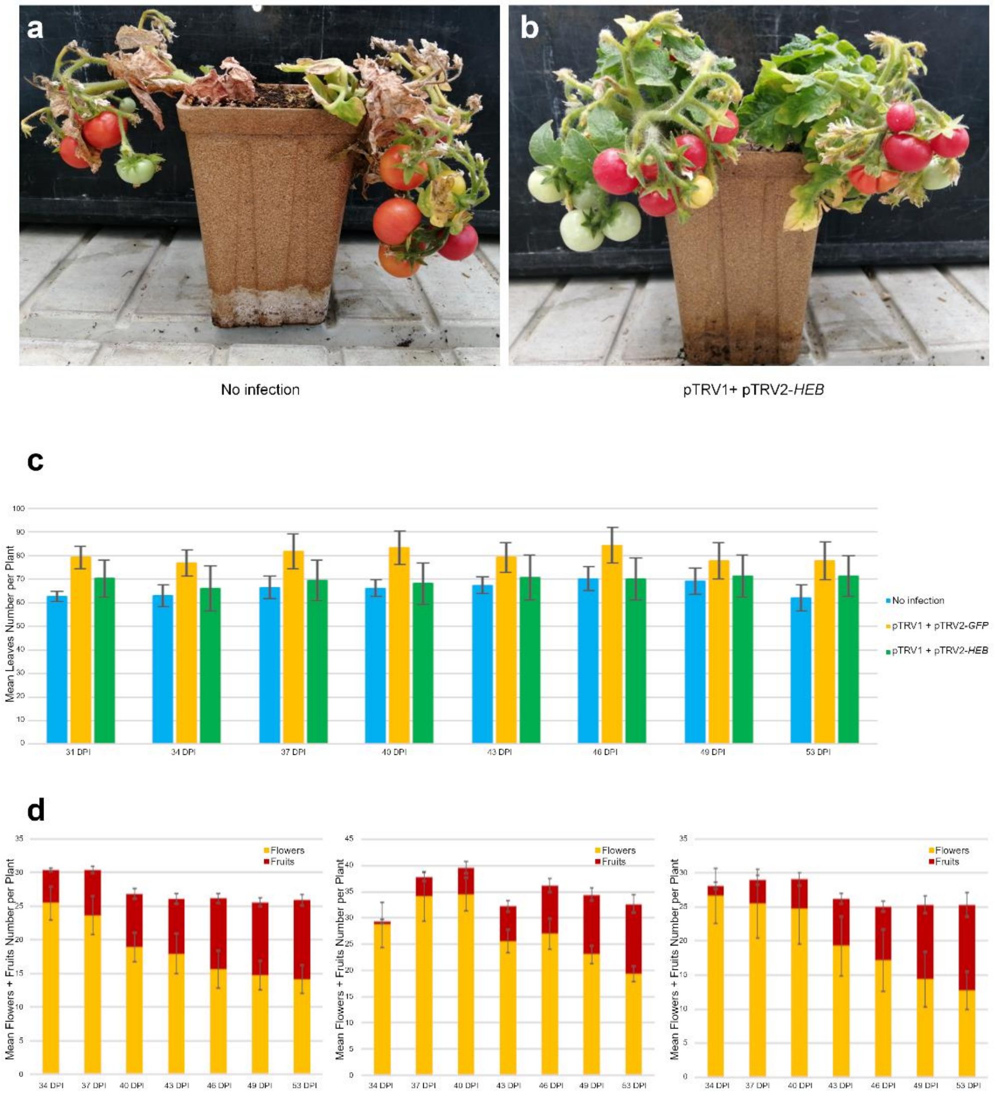

Figure S4. Not infected (**a**) and pTRV1 and pTRV2-*HEB* infected plants (**b**) at 65 dpi. The stay-green phenotype is clearly evident. Beside the stay-green phenotype of pTRV1 + pTRV2-*HEB* infected plants, we could not detect any difference in the number of leaves (**c**) nor in flowers and fruits number (**d**). Bars represent the mean of 10 plants per group of treatment and error bars indicate standard error.

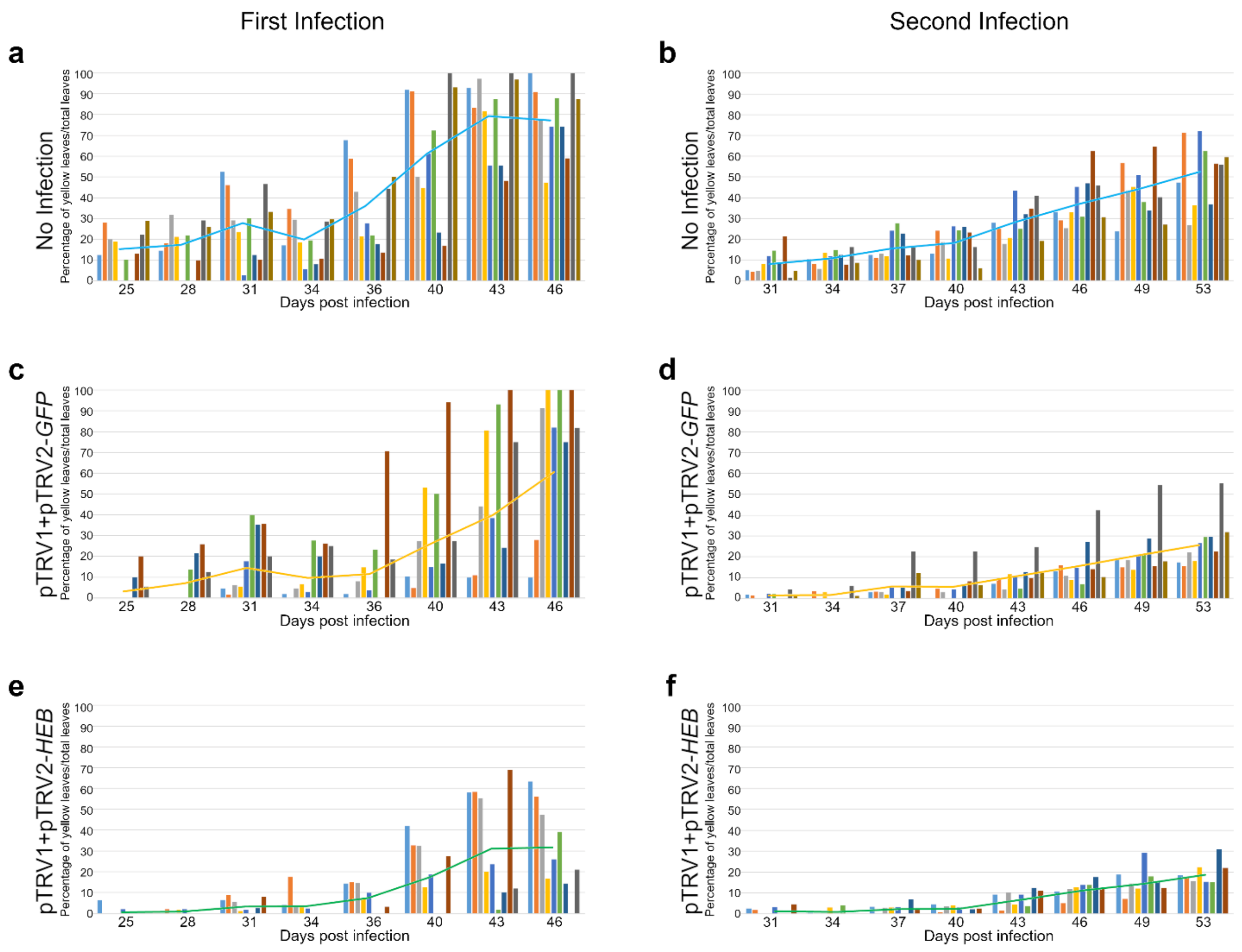

Figure S5. Percentage of yellow leaves on total leaves for not infected plants (**a**,**b**), pTRV1 + pTRV2-*GFP* (**c**,**d**) and pTRV1 + pTRV2-*HEB* (**e**,**f**). Bars represent individual plants for each time point (n=10). Lines represent the mean percentage, as reported in Figure 2c,d.

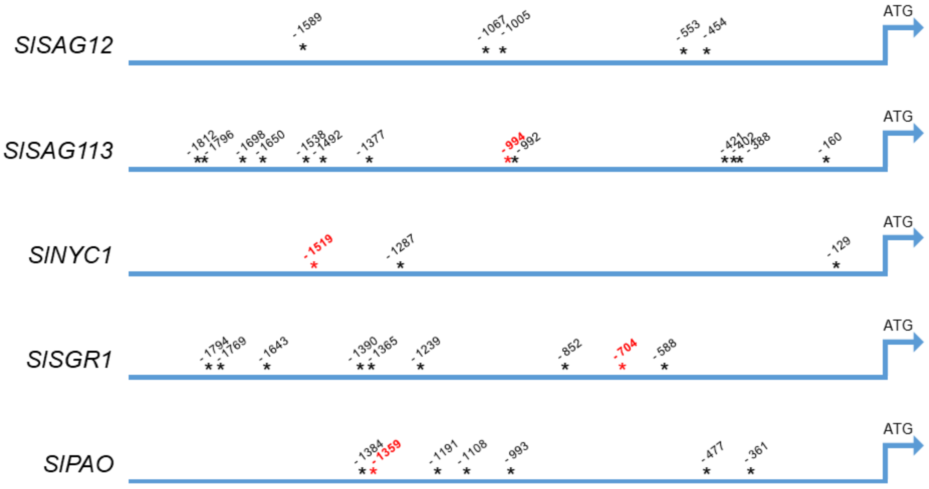

Figure S6. Putative binding sites for NAC transcription factors are listed along the promoter sequence of senescence associated genes (*SlSAG12* and *SlSAG113*) and chlorophyll associated genes (*SlNYC1*, *SlSGR1* and *SlPAO*). In particular, putative AtNAC058 binding sites are highlighted in red.

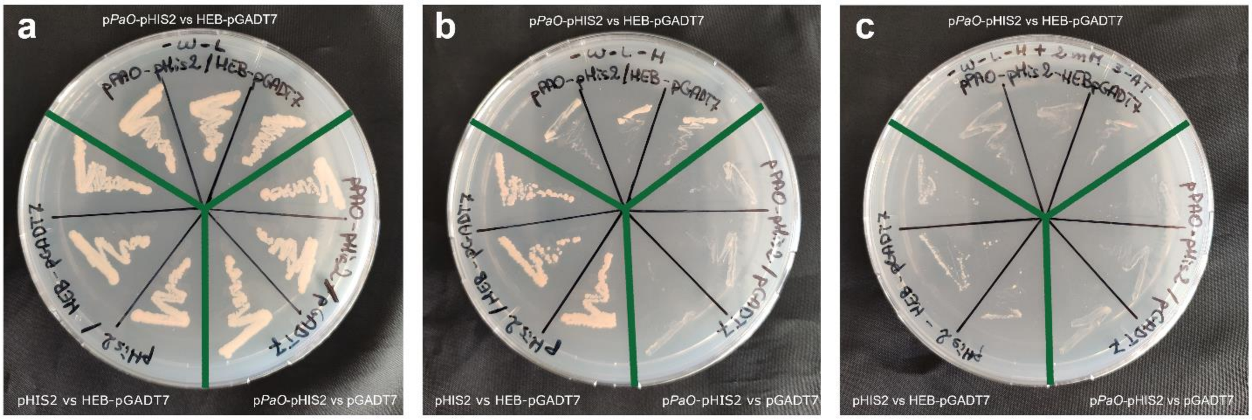

Figure S7. Yeast 1-Hybrid assay was used to test the interaction between HEB and the regulative region of *SlPaO*. Three colonies for the interaction (p*PaO*-pHIS2 vs HEB-pGADT7) and three colonies for each control (pHIS2 vs HEB-pGADT7 and p*PaO*-pHIS2 vs pGADT7) were grown on permissive medium (**a**), selective medium lacking histidine (**b**) and lacking histidine with 2mM of 3AT (**c**).

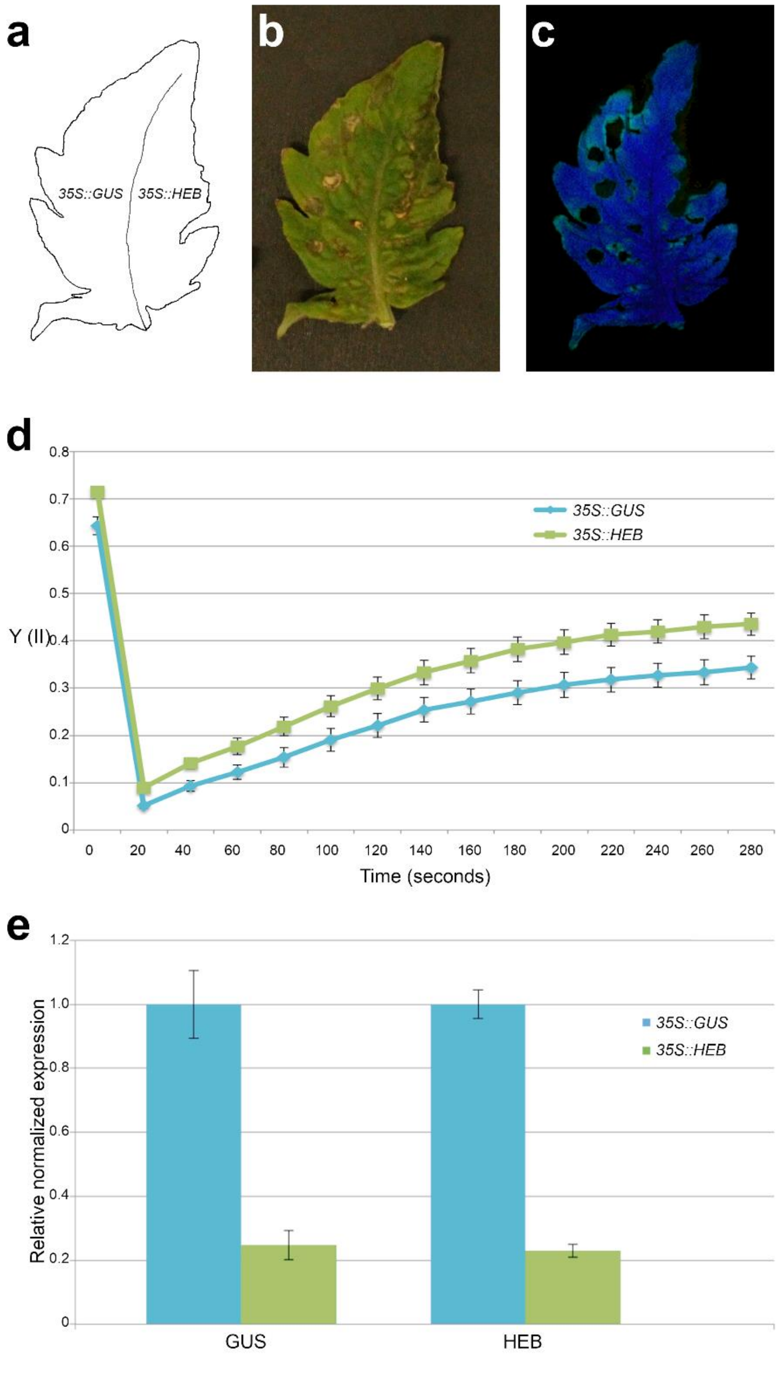

Figure S8. (**a**) Visual scheme of leaves portioning for the infection. (**b**) Picture of a representative leaf at 10 dpi. (**c**) Visual aspect and PSII quantum yield (Fv/Fm, Imaging PAM) of 35S::*GUS* and 35S::*HEB* infected leaves at 10 dpi. (**d**) Measurement of Y(II) in 35S::*GUS* and 35S::*HEB* half leaves. Dots represent the average of 3 technical replicates and error bars indicate standard error. Statistical differences between 35S::*GUS* and 35S::*HEB* was assessed with Tukey HSD test at $P \leq 0.05$. A representative result from two independent experiments is shown. (**e**) qRT-PCR performed on *GUS* and *HEB* transcript in 35S::*GUS* and 35S::*HEB* half leaves. *HEB* transcript is not accumulated in the 35S::*HEB* infected region of the leaves. Bars represent the average of three technical replicates and error bars indicate standard deviation. Three independent replicates were performed and a representative experiment is shown.

Supplemental Table S1 – Primers used in this work

Primers used for qRT-PCR:

Genes	Allele	Primer sequence	Reference
UBI	For	TCGTAAGGAGTGCCCTAATGCTGA	Lacerda et al., 2015
	Rev	CAATCGCCTCCAGCCTTGTTGTAA	
EF1α	For	GATTGACAGACGTTCTGGTAAGGA	Lacerda et al., 2015
	Rev	ACCGGCATCACCATTCTTCA	
HEB	For	ACAACGACTCATGACTACGC	
	Rev	GTTGCTGTTGTTGTTGCTGC	
Solyc06g069100	For	TTATACAACACGGTCGCGGCC	
	Rev	CCTTGAAATCACGCGGATGCG	
SAG12	For	GGTAGTAAATGGGGTGAAAATG	Ma et al., 2018
	Rev	TTAGGCAGTGGGATAAGAAGC	
SAG113	For	AAATGATATTACGGTGACCGGC	Ma et al., 2018
	Rev	CTCAAATCCACCACAACAACAC	
NYC1	For	CCTAACCGACCTACTTCTGAGTGG	Ma et al., 2018
	Rev	AGCAACTGTCTCTGGATGTTCGC	
SGR1	For	CCAGTGAGTGTTATGCCTTGG	Ma et al., 2018
	Rev	TCAACTTTGCTGCTCTTGCAAG	
PAO	For	CATGGAGAACGTCTCTGATCCTTC	Ma et al., 2018
	Rev	TGTCTCGCCTTCCAGTAACCTTG	

Primers used for construct preparation:

Genes	Allele	Primer sequence
HEB for VIGS fragment	For	GGGGACAAGTTTGTACAAAAAAGCAGGCTTCAATTGAAACAACATTTGGTTAC
	Rev	GGGGACCACTTTGTACAAGAAAGCTGGGTACGGTGCCCCAATATTATTC
GFP for VIGS fragment	For	GGGGACAAGTTTGTACAAAAAAGCAGGCTCCATGGTGAGCAAGGGCGAG
	Rev	GGGGACCACTTTGTACAAGAAAGCTGGGTTTACTTGTACAGCTCGTCCATG

HEB for transient expression	For	GGGGACAAGTTTGTACAAAAAAGCAGGCTTCATGGATGAAAATCTTCCTCC
	Rev	GGGGACCACTTTGTACAAGAAAGCTGGGTCTCACCAAATATTGGAGTCC
GUS for transient expression	For	GGGGACAAGTTTGTACAAAAAAGCAGGCTATGTTACGTCCTGTAGAAACCCC
	Rev	GGGGACCACTTTGTACAAGAAAGCTGGGTTCATTGTTTGCCTCCCTGCTGCGG
p*PaO* for Y1H	For_*Eco*RI	GCGAATTCATCTTTCTTGATTAGATATTAG
	Rev_*Eco*RI	GCGAATTCTATTGAGTTAATCAATGTTTTG
